# Tannin extracted from *Penthorum chinense* Pursh, a potential drug with antimicrobial and antibiofilm effects against methicillin-sensitive *Staphylococcus aureus* and methicillin-resistant *Staphylococcus aureus*

**DOI:** 10.3389/fmicb.2023.1134207

**Published:** 2023-07-03

**Authors:** Junyuan Qin, Lei Yu, Fu Peng, Xin Ye, Gangmin Li, Chen Sun, Fang Cheng, Cheng Peng, Xiaofang Xie

**Affiliations:** ^1^State Key Laboratory of Southwestern Chinese Medicine Resources, School of Pharmacy, Chengdu University of Traditional Chinese Medicine, Chengdu, China; ^2^Department of Pharmacology, Key Laboratory of Drug-Targeting and Drug Delivery System of the Education Ministry, Sichuan Engineering Laboratory for Plant-Sourced Drug and Sichuan Research Center for Drug Precision Industrial Technology, West China School of Pharmacy, Sichuan University, Chengdu, China; ^3^Department of Pharmacy, The Affiliated Traditional Chinese Medicine Hospital of Southwest Medical University, Luzhou, China

**Keywords:** Tannin, *Penthorum chinense* pursh, *Staphylococcus aureus*, methicillin-resistant *Staphylococcus aureus*, bacterial biofilm

## Abstract

*Staphylococcus aureus* is an opportunistic pathogen. Due to the widespread use and abuse of antibiotics, various drug-resistant strains of *S. aureus* have emerged, with methicillin-resistant *Staphylococcus aureus* (MRSA) being the most prevalent. Bacterial biofilm is a significant contributor to bacterial infection and drug resistance. Consequently, bacterial biofilm formation has emerged as a therapeutic strategy. In this study, the chemical constituents, antimicrobial and antibiofilm properties of tannins isolated from *Penthorum chinense* Pursh (TPCP) were investigated. *In vitro*, TPCP exhibited antimicrobial properties. The minimum inhibitory concentrations (MIC) and minimum bactericidal concentrations (MBC) for methicillin-sensitive *Staphylococcus aureus* (MSSA) and MRSA were 156.25 and 312.5 μg/mL, and 312.5 and 625 μg/mL, respectively. According to the growth curves, TPCP significantly inhibited the growth of MSSA and MRSA. The results of the crystal violet biofilm assay in conjunction with confocal laser scanning and scanning electron microscopy demonstrated that TPCP destroyed preformed MSSA and MRSA biofilms. TPCP significantly decreased the secretion of exopolysaccharides and extracellular DNA. Subsequently, the mechanism was investigated using RT-PCR. Examining the expression of *ica*A, *cid*A, *sig*B, *agr*A, and *sar*A genes in MRSA, we discovered that TPCP inhibited biofilm formation by affecting the quorum-sensing system in bacteria. Our study demonstrates that TPCP exerts antibacterial effects by disrupting the formation of bacterial biofilms, suggesting that TPCP has clinical potential as a novel antibacterial agent for the prevention and treatment of MSSA and MRSA infections.

## 1. Introduction

*Staphylococcus aureus* is a clinical pathogen that can cause a variety of infectious illnesses, including pericarditis, meningitis, and septicemia (Wu et al., 2021). Prior to the discovery of antibiotics, over 80% of patients with *S. aureus* bacteremia died (Skinner and Keefer, 1941). The widespread application of antibiotics in clinical settings has significantly reduced the proportion of infections-related deaths. However, some strains of *S. aureus* appear to be resistant to antibiotics, such as β-lactam, glycopeptides, lipoglycopeptides, and oxazolidinones (Mlynarczyk-Bonikowska et al., 2022). Methicillin-resistant *Staphylococcus aureus* (MRSA) is the most prevalent strain of drug-resistant bacteria (Guo et al., 2020). MRSA infections account for at least 25–50% of *S. aureus* infections (Diekema et al., 2001). In 2021, the MRSA detection rate in 51 hospitals in major regions of China reached 30% (Hu et al., 2022). Compared to methicillin-sensitive *staphylococcus aureus* (MSSA), MRSA has a much higher rate of antibiotic resistance. The rate of antibiotic resistance in MRSA is substantially higher. Currently, vancomycin is used as a last-resort therapy for severe MRSA infections (Rodvold and Mcconeghy, 2014). Additionally, MRSA is resistant to the majority of antibiotics (Diekema et al., 2001). MRSA is resistant to a variety of antibiotics, including β-lactams, tetracyclines, aminoglycosides, fluoroquinolones, clindamycin, trimethoprim-sulfamethoxazole, vancomycin, daptomycin, and linezolid. Bacteria biofilms are present on the majority of clinically isolated MRSA strains (Cha et al., 2013). Bacterial biofilms increase bacterial resistance to antibacterial drugs, and research findings show that bacteria in biofilms have 10–1,000 times the resistance of planktonic bacteria (Römling and Balsalobre, 2012; Lu et al., 2014). Bacteria biofilm-related resistance mechanisms are multifaceted, including osmotic restriction, external antibiotic pumping systems, resistance gene delivery, nutrient restriction, and biofilm phenotype (Schilcher and Horswill, 2020). Therefore, combating bacteria biofilms is a novel antimicrobial agent discovery strategy.

In ancient China, medicinal plants were the main way to treat infectious diseases (Ma et al., 2019). Nowadays, many Chinese herbs are found to have antibacterial properties through multiple mechanisms, including the inhibition of bacterial biofilm formation and biofilm destruction (Liu et al., 2022). *Penthorum Chinense* Pursh (PCP), also known as Ganhuangcao, Chegencai, or Shuizelan, is a long-term folk herb used by the Miao ethnic minority in China (Ding et al., 2019). Different components are found in PCP, such as phenols, flavonoids, tannin, organic acids and coumarins, in which flavonoids are considered as the active ingredients of PCP. It is proved that PCP has extensive pharmacological effects, including anti-alcoholic fatty liver, anti-non-alcoholic fatty liver, skin protection, antiviral infection, and so on (Wang et al., 2020). The decoction and extract of PCP have been reported to inhibit the growth of *S. aureus in vitro* (Lei et al., 2012; Yu et al., 2017; Ding et al., 2019). In a preliminary study, we discovered that the PCP decoction had a remarkable antibacterial action on *S. aureus* and MRSA and could inhibit bacterial biofilm formation (Qin et al., 2023). The tannin of *Penthorum chinense* Pursh (TPCP) is also active constituents of PCP (Zhang et al., 2017). However, its effect on bacteria is still unclear. Based on the formal finding, in this study, we analyze the chemical composition of TPCP. Then both the MSSA and MRSA are applied to observe antibacterial effect of TPCP *in vitro*, in which the antibacterial and antibiofilm properties of TPCP on MSSA and MRSA were evaluated. The study would not only help to expand the application of PCP, but also provide a potential natural extract as a candidate for MRSA.

## 2. Materials and methods

### 2.1. Extraction of TPCP

Samples of PCP were purchased from the Lotus Pond Chinese herbal medicine market, which was identified by Gao, an associate professor at Chengdu University of Traditional Chinese Medicine. PCP was crushed to a coarse powder and then treated with 50% ethanol 8 times, followed by reflux extraction for 1 h twice. The concoction was concentrated to a relative density of 1.04–1.06 at 60°C. After centrifugation, the supernatant was subjected to chromatography for 2 h on a D-101 macroporous adsorbent resin column. The column was successively eluted with clean water and 70% (*v/v*) EtOH, then the eluate was concentrated at 60°C. Using UV spectroscopy, the tannic acid concentration in TPCP was determined. The standard curve equation was y=6.3201x−0.0089 (*R*^2^ = 0.9989), and the tannic acid concentration was 69.2%. Pure water was used to dissolve the extracts.

#### 2.1.1. UPLC-Q-Orbitrap HRMS analysis

To identify the chemical components of the TPCP, ultra-high-performance liquid chromatography coupled with hybrid quadrupole Orbitrap high-resolution mass spectrometry (UPLC-Q-Orbitrap HRMS) was performed using Vanquish UHPLC-Q Exactive Plus (Thermo Fisher Scientific Inc., MA, United States). The chromatographic column used was a Thermo Scientific Accucore™C_18_ (3 mm × 100 mm, 2.6 μm). With gradient elution, a combination of 0.1% (*v/v*) formic acid solution (A) and 0.1% (*v/v*) formic acid–acetonitrile solution (B) served as the mobile phase. The gradient program was as follows: 0–9 min, 5–17% B; 9–17 min, 17–30% B; 17–25 min, 30–50% B; 25–30 min, 50–80% B; 30–35 min, 80–90% B; and 35–40 min, 99% B. The flow rate was 1.0 mL/min, and the column temperature was set at 35°C. The injection volume of the sample (0.8 mg/mL, *w/v*) was 3 μL. For positive ion-mode electrospray ionization, the following MS settings were used: capillary voltage, 3.2 KV; sheath gas, 35 arb; auxiliary gas, 10 arb; capillary temperature, 320°C; full millisecond resolution, 70,000; MS^2^ resolution, 17,500; normalized collision energy, 20/40/60 eV; scanning range, m/z 100–1,500. Discoverer software (version 3.0) was used for data collection and processing. The measured spectra of the secondary fragments were matched with the mzCloud network database and OTCML, a local Chinese medicinal composition database, using the following parameters: peak area threshold, 80,000; mass deviation, 5 ppm; a match score greater than 80.

#### 2.1.2. High-performance liquid chromatography

An Agilent Technologies 1,290 Infinity instrument (Agilent Technologies (China) Co. Ltd., China) was used for HPLC analysis. Octadecyl silane chemically bonded to silica (Ultimate LP-C18, 150 × 4.6 mm, 5 μm) was selected as the chromatographic column. A column temperature of 30°C and an aqueous solution of acetonitrile/0.1% (*v/v*) phosphoric acid water isocratic mobile phase (0 min, 10:90; 45 min, 35:65) were used. Column elution was determined using a UV detector at 280 nm.

The dried powders of the TPCP samples (18 mg) were precisely weighed and dissolved in 10 mL 100% methanol solution. Tannic acid (CAS 1401-55-4, RENI, Chengdu, China), epicatechin (CAS 490–46-0, RENI, Chengdu, China), and ellagic acid (CAS 476–66-4, RENI，Chengdu, China) were precisely formulated in 0.114, 0.10, and 0.098 mg/mL(*w/v*) methanol solutions, respectively. After passing through ultrasonic (150 W, 40 kHz) and a 0.22-m filter membrane, all solutions were added to the original weight.

### 2.2. Strains and culture conditions

MSSA was provided by the National Institutes for Food and Drug Control (CMCC, Beijing, China) and MRSA was provided by the China Center for Type Culture Collection (CCTCC, Wuhan, China). Both MSSA and MRSA strains were incubated with nutrient agar for 16–18 h at 35°C. The turbidity of a 0.5 McFarland standard (1.5 × 10^8^ CFU/mL) was used to adjust the inoculum. Bacterial cultures were diluted 1:30 with test medium to give a working inoculum of 5 × 10^6^ CFU/mL.

### 2.3. MIC and MBC tests

The trace broth microdilution method is recommended for MIC determination according to the Clinical and Laboratory Standards Institute (CLSI, 2021). However, the TPCP solution is dark in color and would interfere with the results by this method. To solve this problem, we finally performed the MIC measurement with combination of the trace broth microdilution method and the 2,3,5-triphenyl-2H-tetrazolium chloride (TTC) staining (Veiga et al., 2019). First, 100 μL of bacterial solution was added in each well of a 96-well plate which was added with 100 μL per well of 78.125 μg/mL TPCP solution in advance. The plate was put in incubator for 24 h, then 0.25%(*v/v*) TTC (Sigma-Aldrich, United States) was added to each well and incubated for 30 min. Macroscopically, the lowest concentration that did not turn red was defined as the MIC. 100 μL of the supernatant was collected, inoculated onto an MHA plate, and cultured for 24 h. The minimum bactericidal concentration (MBC) is defined as the lowest drug concentration that kills more than 99.9% of bacteria. We have judged the MBC to be less than 5 colonies based on the reference (Guang et al., 2015; Rodríguez et al., 2021). The susceptibilities of MRSA and MSSA to vancomycin (Solarbio, Beijing, China) and oxacillin sodium salt (Aladdin, Shanghai, China) were evaluated using the same method. The experiment was repeated three times, with three parallel samples each time.

### 2.4. Time-killing growth curve assay

The bacterial suspension (2 mL) was cultured in 12-well plates for 24 h at 35°C and 120 rpm with varying final concentrations of TPCP (2×, 1×, 1/2×, and1/4 × MIC). A control group was set up separately. Viable colony counts in each well were determined at 0, 4, 8, 16, and 24 h using the spread plate count method. MSSA and MRSA time-kill curves were plotted with CFU/mL as the ordinate and time as the abscissa (Wang et al., 2021). The experiment was repeated three times with three parallel samples each time.

### 2.5. Biofilm assay by crystal violet

MSSA and MRSA suspensions (1.5 × 10^8^ CFU/mL) were incubated in 96-well polystyrene microplates at 35°C for 48 h (Haney et al., 2021). Cultures of TPCP (2×, 1 × and 1/2 × MIC) were incubated in 96-well plates at 35°C for 24 h. A control group was set up separately. PBS was used to wash away residual medium. After methanol fixation, 150 μL of 0.1% (v/v) crystal violet solution (Solarbio, Beijing, China) was added to the 96-well plates for 15 min, then washed with tap water and dried naturally. The samples were then solubilized with 95% (v/v) absolute ethanol, and the OD590nm was measured using the Cytation 5 Cell Imaging Multi-Mode Reader (BioTeK, United States). The experiment was repeated three times with five parallel samples each time.

### 2.6. Confocal laser scanning microscopic assay of biofilm

SYTO9 green fluorescent nucleic acid staining was used to assess the effect of TPCP on the viability of MSSA and MRSA biofilm (Jefferson et al., 2005). Bacterial suspension (2 mL, 1.5 × 10^8^ CFU/mL) was added to a glass-bottomed cell culture dish (NEST, Wuxi, China) and incubated for at 35°C 72 h. The planktonic bacteria were then removed, and 500 μL of fresh MHB containing different final concentrations of TPCP (2×, 1×, and 1/2 × MIC) were added. A control group was set up separately. After 8 h of incubation, the supernatant was discarded and the cells were washed with 5 mM 4-(2-hydroxyethy)-1-piperazine-1-ethanesulfonic acid (HEPES) buffer (pH 7.2). STYO9 (1 μL, Thermo Fisher, USA) was added to each dish and incubated for 20 min, and observed under a CLSM (Olympus FV1200, Japan) after the dye was removed. This experiment was repeated three times.

### 2.7. Scanning electron microscopic assay of biofilm

The bacterial suspension (1.5 × 10^8^ CFU/mL) was incubated in 6-well plates with coverslips at 35°C for 72 h. The culture medium supernatant was aspirated, 3 mL of fresh MHB containing different final concentrations (2×, 1×, and 1/2 × MIC) of TPCP was added, and allowed to stand for 8 h. A control group was set up separately. Cleaning and fixation of coverslips were performed as described previously [9]. Different concentrations (30, 50, 70, 90, and 100%, *v/v*) of ethanol were then used for the dehydration gradient. Freeze-drying (Eyela FDI-2110, Shanghai, China) was used as the drying method, and the dried samples were sprayed with gold and evaluated by SEM (ZEISS EVO10, Germany).

### 2.8. Extracellular exopolysaccharides content assay

The bacterial suspension was incubated with different final concentrations (1×, 1/2×, and 1/4 × MIC) of TPCP for 24 h at 35°C. A control group was set up separately. EPS extraction was performed as previously described (Li et al., 2020). The EPS content was detected by the phenol-sulfuric acid method at 490 nm using D (+)-dextrose anhydrate (Solarbio, Beijing, China) as a standard. The equation of the standard curve was y=0.10756+4.45949x (*R*^2^ = 0.9969). The experiment was repeated three times with three parallel samples each time.

### 2.9. Extracellular DNA assay

The bacterial suspension was incubated at 35°C in 12-well plates with varying concentrations of TPCP (1×, 1/2×, and 1/4 × MIC). A control group was set up separately. The extraction of eDNA was performed as previously described (Rice et al., 2007). The OD600nm values of the samples were measured using a Cell Imaging Multi-Mode Reader (BioTeK Cytation 5, United States). The experiment was repeated three times with three parallel samples each time.

### 2.10. Quantitative real-time PCR (RT-PCR) analysis

After incubation of MRSA suspensions with TPCP (1× MIC) for 8 and 16 h, total RNA was extracted by using the Trizol method (Ambion, USA). A control group was set up separately. Total RNA was reverse-transcribed into cDNA at approximately 1 μg RNA concentration using RevertAid Master Mix and DNase I (Thermo Fisher Scientific, USA). The expressions of biofilm-related (*ica*A, *agr*A, *sar*A, and *sig*B) and eDNA-related genes (*cid*A) were tested by RT-PCR using TB Green Premix Ex Taq (Tli RNaseH Plus) Kit (Takara, Japan). *gy*rB was used as the reference gene and the results were analyzed by 2^-△△Ct^ method. The experiment was repeated three times. The primers used are listed in Table 1.

**Table 1:** Primers used for all of the studies.

Name	Sequence (5′ to 3′)	References
*cid*A Forward	TGTACCGCTAACTTGGGTAGAAGAC	Jin et al. (2020)
*cid*A Reverse	CGGAAGCAACATCCATAATACCTAC	
*agr*A Forward	TGATAATCCTTATGAGGTGCTT	Wang et al. (2021)
*agr*A Reverse	TGT TCGTAACGAAAA	
*ica*A Forward	CTGGCGCAGTCAATACTATTTCGGGTGTCT	Wang et al. (2021)
*ica*A Reverse	GACCTCCCAATTTTCTGGAACCAACATCC	
*sar*A Forward	CAAACAACCACAAGTTGTTAAAGC	Wang et al. (2021)
*sar*A Reverse	TGTTTGCTTCAGTGATTCGTT T	
*sig*B Forward	AAG TGA TTC GTA AGG ACG TCT	Wang et al. (2021)
*sig*B Reverse	AAG TGA TTCGTAAGGAC TCT	
*gyrB* Forward	GGTGGCGACTTTGATCTAGC	Otto et al. (2013)
*gyrB* Reverse	TTATACAACGGTGGCTGTGC	

## 3. Results

### 3.1. Chemical constituents of TPCP

**Figure 1:**
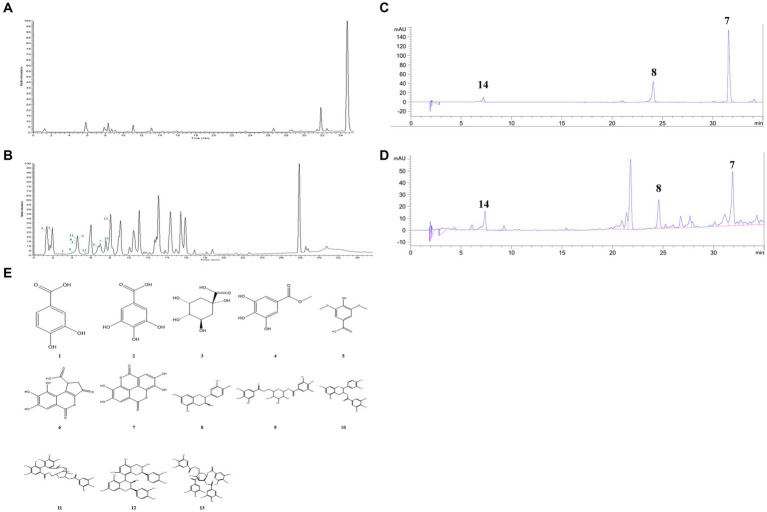
Chemical constituents of TPCP. **(A)** and **(B)** UPLC-Q-Orbitrap HRMS base peak chromatogram of **(A)** positive and **(B)** negative modes; **(C)** Standard components of TPCP detected by HPLC, which include ellagic acid (7), epicatechin (8), and tannic acid (14); **(D)** Ellagic acid (7), epicatechin (8), and tannic acid (14) were detected in the TPCP extract by HPLC; **(E)** Chemical structures of TPCP were illustrated which according to UPLC-Q-Orbitrap HRMS and HPLC.

The components of TPCP were separated using the optimal UPLC-Q-Orbitrap HRMS conditions, and the chromatograms of the base peaks obtained in positive and negative ionization modes are shown in Figures 1A,B. As shown in Table 2, TPCP was determined to contain 3,4-dihydroxybenzoic acid (**1**), gallic acid (**2**), quinic acid (**3**), methyl gallate (**4**), ethyl gallate (**5**), brevifolincarboxylic acid (**6**), ellagic acid (**7**), epicatechin (**8**), digalloyl-glucose (**9**), (−)-epicatechin gallate (**10**), corilagin (**11**), procyanidin B1(**12**), and tetragalloyl-glucose (**13**). Their information and structures are shown Figure 1E and Table 1. In the HPLC assay, we detected tannic acid (**14**), which was not detected by UPLC-Q-Orbitrap HRMS. The tannic acid, epicatechin, accounted for 14.31, 1.34, and 0.72% of the TPCP mass, respectively (see Figures 1C,D).

**Table 2:** Analysis of TPCP using UPLC-Q-Orbitrap HRMS.

NO.	Retention time (min)	Ionization mode	Molecular formula	Theoretical (m/z)	Measured (m/z)	Error (ppm)	MS/MS Fragments (m/z)	Identified compounds	References
1	2.97	[M-H]^−^	C_7_H_6_O_4_	154.0266	153.01889	−2.87	109.0287,108.0209,80.0259	3,4-Dihydroxybenzoic acid	Vega-Ruiz et al. (2021)
2	1.96	[M-H]^−^	C_7_H_6_O_5_	170.0215	169.0139	−2.25	169.0138,125.0237,95.01230,69.0336	Gallic acid	Nijat et al. (2020)
3	1.33	[M-H]^−^	C_7_H_12_O_6_	192.0634	191.0558	−1.13	191.0558,111.0082,87.0442,85.02860,59.0128	Quinic acid	Nijat et al. (2020)
4	4.36	[M-H]^−^	C_8_H_8_O_5_	184.0372	183.0296	−2.1	183.0295,154.9983,124.01594,109.9999,83.28570	Methyl gallate	Pekacar and Deliorman (2022)
5	6.79	[M-H]^−^	C_9_H_10_O_5_	198.0528	197.0453	−0.96	168.0060,125.0237,110.0000,67.01778	Ethyl gallate	Zheng et al. (2021)
6	4.61	[M + H]^+^	C_13_H_8_O_8_	292.0219	293.0296	1.56	247.0242,219.0291,191.0341,105.1271	Brevifolincarboxylic acid	Liu et al. (2019)
7	6.98	[M-H]^−^	C_14_H_6_O_8_	302.0063	300.9994	1.56	300.9993,283.9966,257.0089,245.0091,229.0143	Ellagic acid	Balkrishna et al. (2022)
8	4.47	[M-H]^−^	C_15_H_14_O_6_	290.0790	289.0724	1.99	289.0711,245.0821,203.0707,151.0395,109.0287	epicatechin	Nijat et al. (2020)
9	4.13	[M-H]^−^	C_20_H_20_O_14_	484.0853	483.0788	1.82	483.0781,331.0668,271.0462,169.0137,125.0236	Digalloyl-glucose	Graça et al. (2016)
10	7.57	[M-H]^−^	C_22_H_18_O_10_	442.0900	441.0832	1.47	289.0721,245.0816,203.0709,125.0237,97.0286	(−)-Epicatechin gallate	Jeong et al. (2014)
11	4.54	[M-H]^−^	C_27_H_22_O_18_	634.0806	633.0742	1.22	633.0743,463.0509,300.9993,257.0091,123.0082	Corilagin	Ismail et al. (2020)
12	5.20	[M-H]^−^	C_30_H_26_O_12_	578.1424	277.1357	2.24	533.0943,451.1034,407.0772,289.0723,125.0237	Procyanidin B1	Jeong et al. (2014)
13	8.03	[M-H]^−^	C_34_H_28_O_22_	788.1072	787.1008	1.20	635.0868,617.0791,465.0677,313.0571,169.0138	Tetragalloyl-glucose	Graça et al. (2016)

### 3.2. MIC and MBC of TPCP on MSSA and MRSA

**Figure 2:**
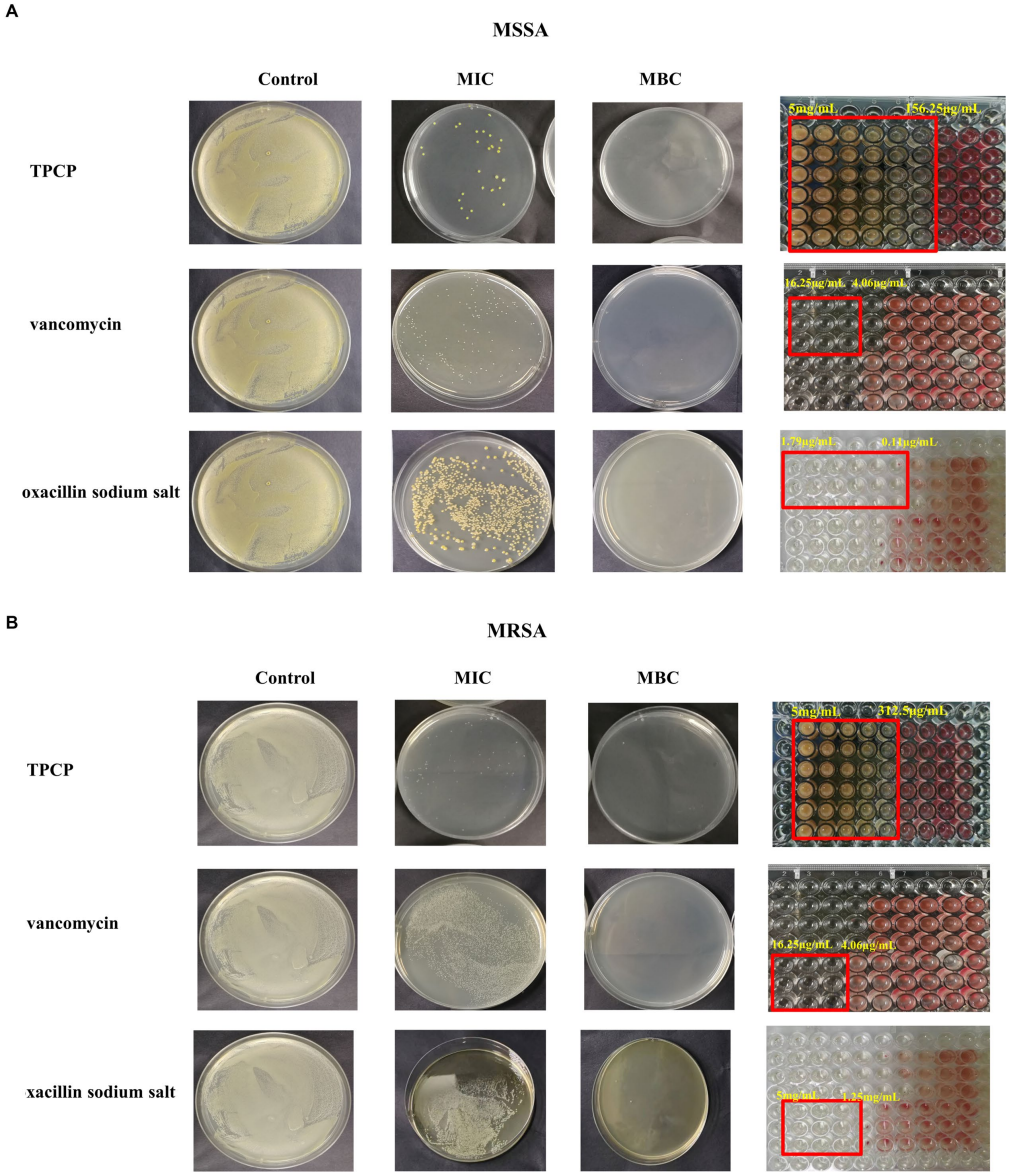
The MIC and MBC values of TPCP for MSSA and MRSA. **(A)** Analysis of the antibacterial activity of TPCP against MSSA using the double dilution and plate-smearing methods; **(B)** Analysis of the antibacterial activity of TPCP against MRSA using the double dilution and plate-smearing methods.

TPCP inhibited the growth of both MSSA and MRSA *in vitro*. The MIC and MBC of TPCP for MSSA were 156.25 and 312.5 μg/mL, respectively. For MRSA, the MIC and MBC of TPCP were 312.5 and 625 μg/mL, respectively. According to CLSIM, the MRSA was resistant to oxacillin sodium salt (MIC:1.25 mg/mL) and susceptible to vancomycin (MIC: 4.06 μg/mL), whereas MSSA was susceptible to both. The growth of MSSA and MRSA was significantly suppressed in the TPCP groups within 24 h of dosing (Figure 2). The growth curves of both MSSA and MRSA showed significant decrease in bacterial counts in all TPCP-treated groups after 4 h. At 8 and 16 h of administration, 1/4× and 1/2 × MIC showed an upward trend in the number of MSSA and MRSA, whereas number of bacteria in 1× and 2 × MIC continued to decrease. After 24 h of administration, when the TPCP concentration was 1 × MIC, the numbers of viable MSSA and MRSA in the TPCP (1 × MIC) group decreased to 32 and 26 CFU/mL, respectively, manifesting the bacteriostatic effect. The numbers of viable MSSA and MRSA in the TPCP (MBC) group decreased to 0 and 1 CFU/mL, respectively, indicating sterilization.

### 3.3. Effect of TPCP on mature MSSA and MRSA biofilms

**Figure 3:**
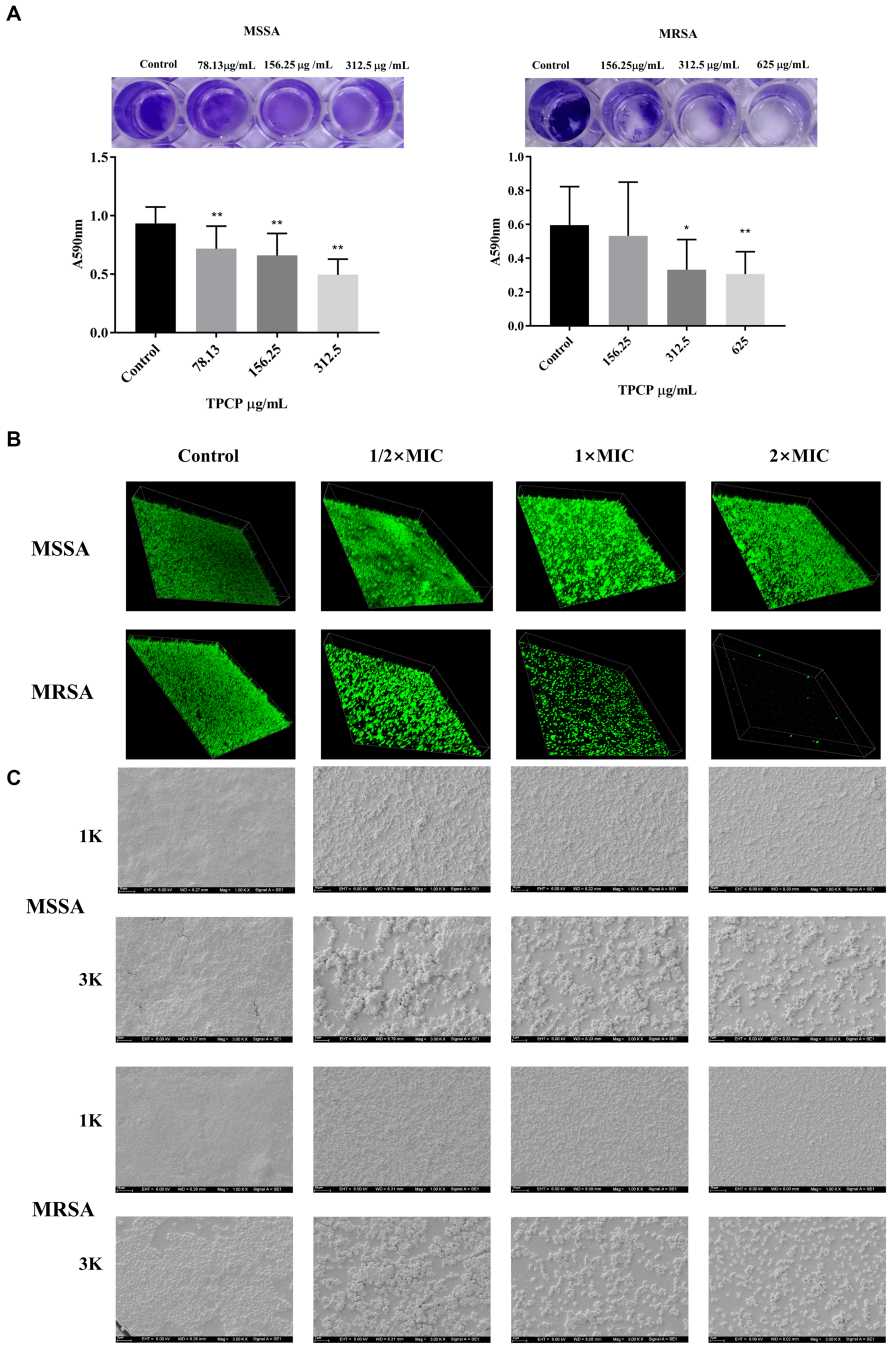
Effect of TPCP on MSSA and MRSA biofilms. **(A)** Crystal violet-stained well plate after TPCP treatment; **(B)** Image of biofilm-formation inhibition activity of TPCP against MSSA and MRSA obtained using confocal laser scanning microscopy, scanning at 100× magnification. **(C)** Image of biofilm-formation inhibition activity of TPCP against MSSA and MRSA obtained using scanning electron microscopy. Scanning electron micrographs at 1.00 and 3.00 k × magnifications, respectively. For MSSA, 2× MIC: 312.5 μg/mL, 1× MIC: 156.25 μg/mL, 1/2 × MIC: 78.13 μg/mL; for MRSA, 2× MIC: 625 μg/mL, 1× MIC: 312.5 μg/mL, 1/2 × MIC: 156.25 μg/mL. All data are presented as the mean values ± SD, and *n* = 15 in each group. ^*^p < 0.05, ^**^p < 0.01, versus the control.

Crystal violet is a basic dye capable of binding negatively charged surface molecules on the surface of the biofilm and polysaccharide components in the extracellular matrix. Therefore, it is often used in research as a preliminary screening for the anti-biofilm ability of drugs (Haney et al., 2021). In this study, we used it to observe the effect of TPCP on MSSA and MRSA biofilms. The results showed a reduction in the area of the biofilm, which consequently caused a decrease in the absorbance of the ethanol solution. The effect became more pronounced as the TPCP dose was increased (*p* < 0.05 or *p* < 0.01, Figure 3A). Bacterial biofilm is a three-dimensional community of bacterial cells that adhere to either biotic or abiotic surfaces. SYTO9 green fluorescent nucleic acids can pass through the bacterial cell walls and membranes and bind directly to the nucleic acids of both living and dead cells. Therefore, the SYTO9 has been used in the CLSM detection of biofilms. The degree of fluorescence staining reflects the number of bacteria and the thickness of the biofilm. The 3D scanning technology of the CLSM captures this fluorescence signal well. The test showed that the biofilms of the TPCP-treated group became thinner and looser compared to the blank group (Figure 3B).The EPS, eDNA, polysaccharides, and lipids provide mechanical stability to bacterial biofilms, immobilizing bacteria and encapsulating them in a viscous matrix to withstand extreme environments and resist antibiotic and host immune responses (Alpkvist et al., 2006; Abebe, 2020). The SEM detection clearly showed that the bacteria in the control group were tightly wrapped in biofilm. Compared with the control group, the biofilm structure of the TPCP-treated groups was destroyed, and the adhesion and aggregation of bacteria was also reduced. In addition, the biofilm structure of MSSA and MRSA was significantly reduced in the TPCP-treated groups, as shown by the SEM images (Figure 3C).

### 3.4. Effect of TPCP on EPS secretion and eDNA of MSSA and MRSA biofilm

**Figure 4:**
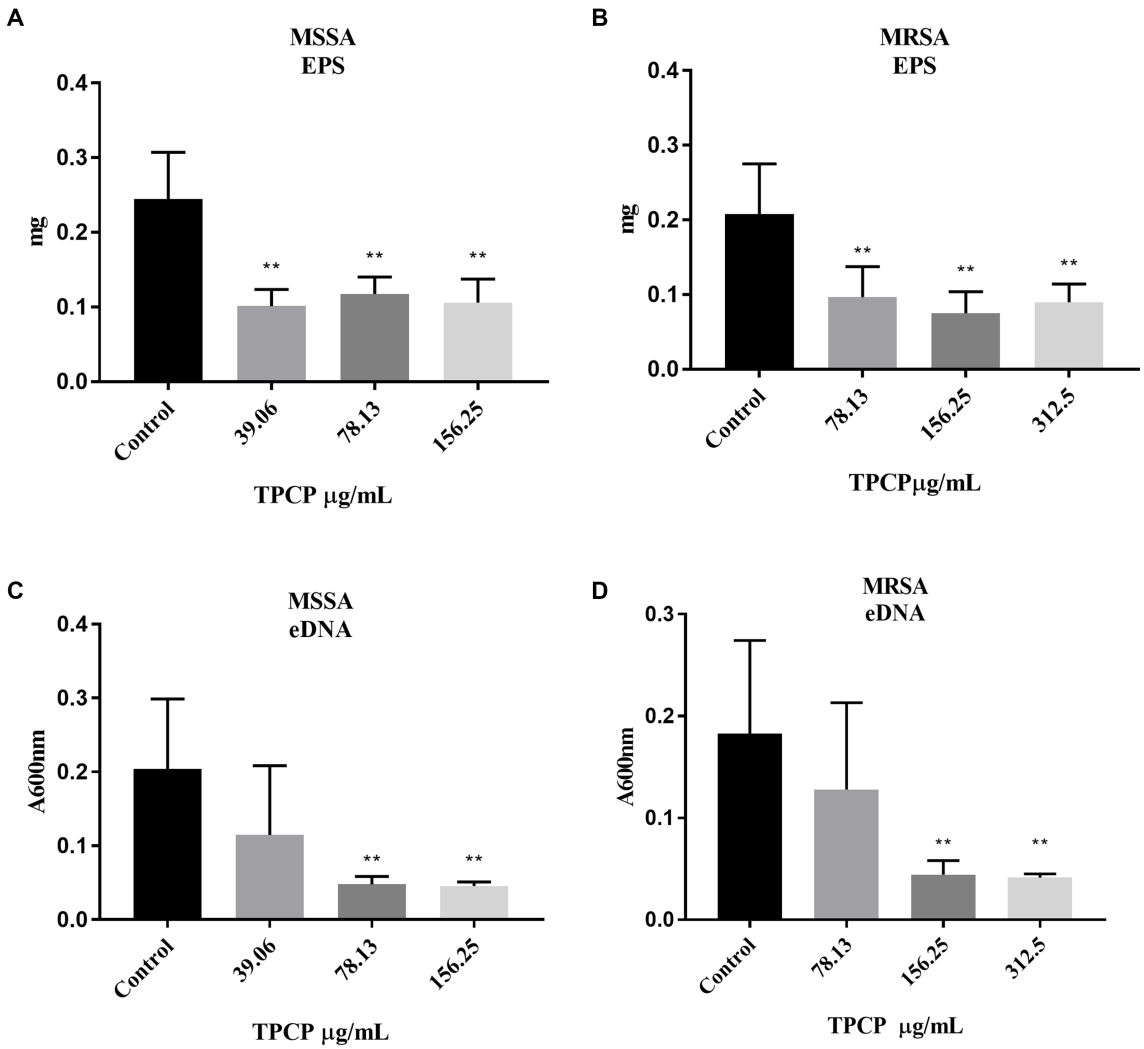
Effect of TPCP on EPS secretion and eDNA of MSSA and MRSA biofilm. **(A)** and **(B)** Effect of TPCP on EPS secretion by MSSA and MRSA biofilm; **(C)** and **(D)** Effect of TPCP on eDNA secretion by MSSA and MRSA biofilm. All data are presented as the mean values ± SD, and n = 9 in each group. ^*^p < 0.05, ^**^p < 0.01, versus the control.

To determine the mechanism of action of TPCP in bacterial biofilm disruption, we examined its effect on EPS and eDNA, which are crucial for biofilm formation. After exposure to TPCP for 24 h, the EPS level in MSSA and MRSA in TPCP-treated groups were all significantly decreased to 39.06, 78.13, and 156.25 μg/mL (*p* < 0.01, Figures 4A,B), among which the EPS level of MRSA in the TPCP (156.25 μg/mL) was the lowest. Besides, TPCP significantly decreased eDNA secretion of *MSSA* and MRSA (*p* < 0.01, Figures 4C,D).

### 3.5. Effect of TPCP on the gene expression of MRSA biofilm-related factors

**Figure 5:**
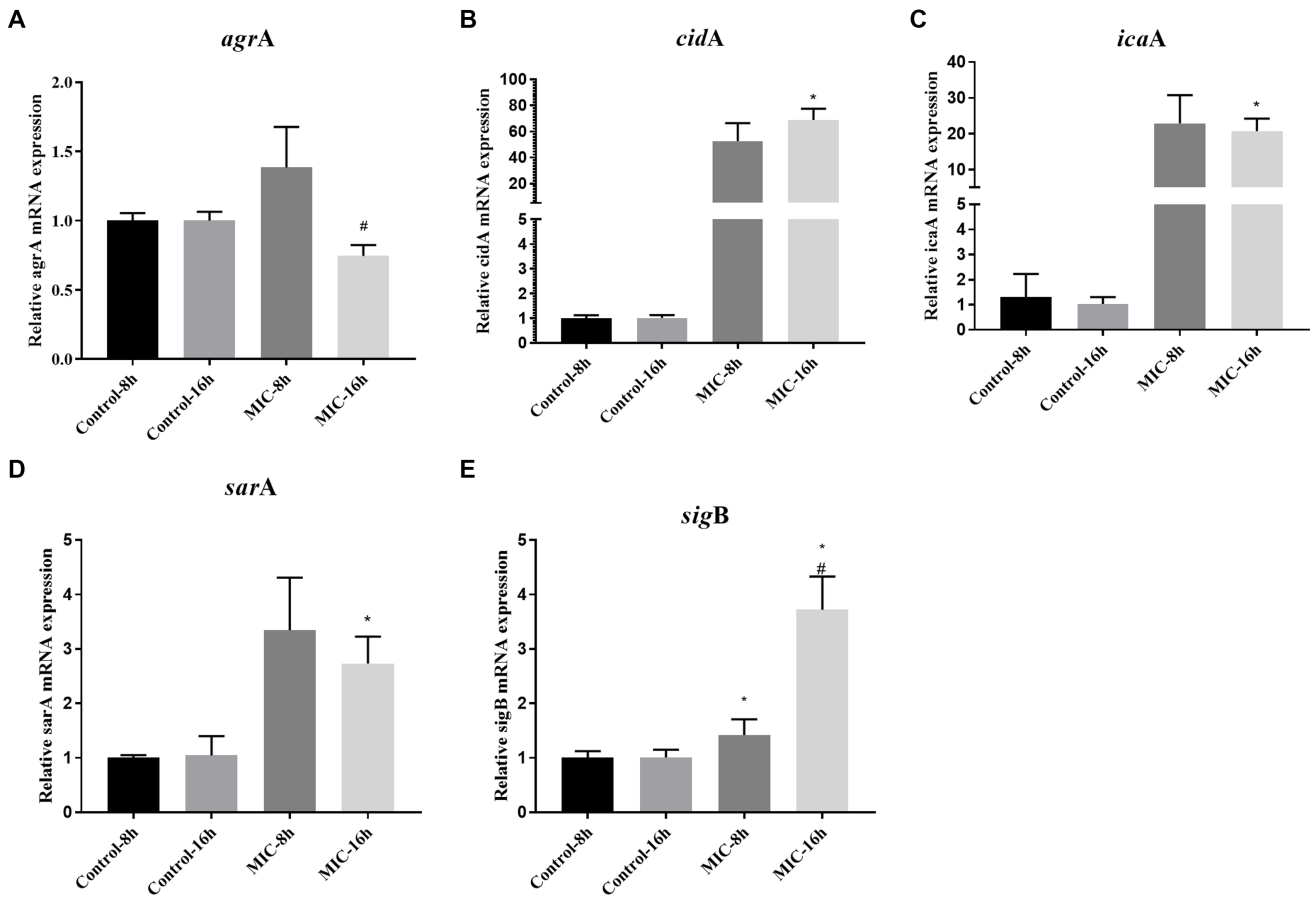
Effect of TPCP on the gene expression of MRSA biofilm-related factors. **(A–E)**: *agr*A, *cid*A, *ica*A, *sar*A *sig*B. All data are presented as the mean values ± SD, and *n* = 3 in each group. ^*^p < 0.05, versus the control; *^#^p* < 0.05, versus the MIC-8 h group.

As shown in Figures 5A–E, after 8 h of administration, TPCP upregulated the expression of the *sig*B gene in MRSA, and the expressions of *agr*A, *ica*A, *sar*A, and *cid*A genes showed an upward trend. At 16 h, the expressions of icaA, sarA, cidA, and sigB genes were significantly increased compared with the control group (*p* < 0.05). Compared with 8 h, the expression of *agr*A gene at 16 h was significantly decreased, while the expression of *sig*B gene was significantly increased in the TPCP-treated group (*p* < 0.05).

## 4. Discussion

Tannins are mainly divided into hydrolyzable and condensed tannins. Hydrolyzable tannins consist of gallic acid, ellagic acid, and other repeating units of polyhydric alcohols. Condensed tannins consist mainly of catechins and anthocyanin aglycones (Fabbrini et al., 2022). To the best of our knowledge, this is the first report of the chemical constituents of TPCP characterized by UPLC-Q-Orbitrap HRMS. The chemical profile of TPCP is similar to that reported previously (Sun, 2016; Yin et al., 2020). To the best of our knowledge, this is the first report on the chemical constituents of TPCP characterized by UPLC-Q-Orbitrap HRMS. The chemical profile of TPCP is similar to that reported previously (Sun, 2016; Yin et al., 2020). In this study, a total of 14 tannin components were successfully detected, including 3,4-dihydroxybenzoic acid (**1**), gallic acid (**2**), quinic acid (**3**), methyl gallate (**4**), ethyl gallate (**5**), brevifolincarboxylic acid (**6**), ellagic acid (**7**), epicatechin (**8**), digalloyl-glucose (**9**), (−)-epicatechin gallate (**10**), corilagin (**11**), procyanidin B1 (**12**), tetragalloyl-glucose (**13**) and tannic acid (**14**). Compounds 1–13 were detected under UPLC-Q-Orbitrap HRMS conditions. Compound **14** was not determined by UPLC-Q-Orbitrap HRMS but was found under HPLC conditions, and its content was 14.3% TPCP. Due to its large molecular weight and small peak area, it may not have been detected under UPLC-Q-Orbitrap HRMS conditions. Tannic acid was the most in TPCP as determined by HPLC analysis. Tannic acid was found to be the most in TPCP by HPLC analysis. It is known to have a strong inhibitory effect on both gram-positive and gram-negative bacteria. Based on this property, biopolymer crosslinkers are often used to prepare antibacterial materials (Kaczmarek, 2020).Recent data indicate that it had an average MIC of 80 μg/mL and was able to destroy bacterial biofilm at 40 μg/mL (Dong et al., 2018). Tannic acid can increase the sensitivity of bacteria to antibiotics (Tintino et al., 2016, 2017; Kırmusaoğlu, 2019). In addition, it reduces bacterial biofilm formation and colonization by inducing the expression of immunodominant staphylococcal antigen A (IsaA) (Payne et al., 2013).

**Figure 6:**
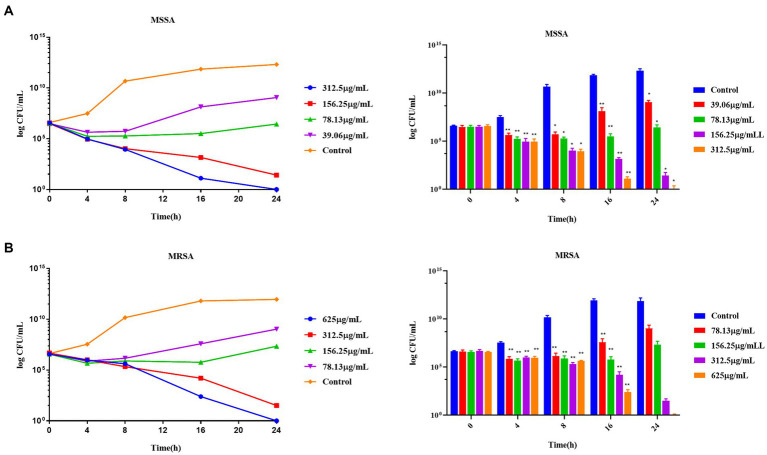
The time-killing curves of TPCP against MSSA and MRSA. **(A)** The growth curve of TPCP against MSSA; **(B)** The growth curve of TPCP against and MRSA. All data are presented as the mean values ± SD, and *n* = 9 in each group. ^*^p < 0.05, ^**^p < 0.01, versus the control.

In this study, the antimicrobial activities of TPCP against *MSSA* and MRSA were evaluated. TPCP exhibited bactericidal activity at relatively low concentrations (156.25 and 312.5 μg/mL, Table 3). The killing curves show the inhibition profile of TPCP against the growth of MSSA and MRSA (Figure 6). TPCP showed superior antibacterial activity and was used in subsequent studies. Absorbance was measured to verify the effects of TPCP on biofilm formation. Crystal violet staining and SEM revealed that TPCP inhibited MSSA and MRSA biofilm formation in a concentration-dependent manner (Figure 3). Furthermore, the mechanism by which TPCP inhibited MRSA biofilm formation at 8 and 16 h was investigated.

**Table 3:** The MIC and MBC values of TPCP for MSSA and MRSA.

Strain		TPCP (μg/mL)	Vancomycin (μg/mL)	Oxacillin sodium salt (μg/mL)
MSSA	MIC	156.25	4.06	0.11
	MBC	312.5	8.13	0.89
MRSA	MIC	312.5	4.06	1,250
	MBC	625	8.13	5,000

Bacterial biofilms promote the adhesion of MRSA to the host or body surface, resulting in persistent infection (Bosch et al., 2020; Cascioferro et al., 2021). The biofilm formation process is divided into four stages: adhesion, microcolony formation, biofilm maturation, and detachment or dispersion (Bai et al., 2021). EPS is a necessary substance in the microcolony formation and maturation stages of biofilm formation (Bai et al., 2021). EPS acts as a framework for bacterial adhesion, increases the viscosity and elasticity of biofilms, and facilitates the exchange of bacterial nutrients and genetic material within biofilms (Vaccari et al., 2017). In our study, the results showed that TPCP significantly inhibited the secretion of EPS (Figures 4A,B). It suggested that the inhibitory effect of TPCP on bacterial biofilms may be associated with microcolony formation and maturation of biofilms.

Deacetylation of the N-acetylglucosamine residue imparts a positive charge to PIA. In addition to electrostatic interaction with charged wall phosphoric acid, PIA binds to various negatively charged substances on bacterial surfaces and negatively charged substrates. This electrostatic action promoted adhesion between bacteria, allowing *S. aureus* to form cohesive and stable biofilms (Formosa-Dague et al., 2016). The products encoded by the intercellular adhesion gene cluster (*ica*) ABCD operon can influence the formation of PIA (O’neill et al., 2008). The sequences of *ica*A and N-acetylglycosaminyltransfer generated by *ica*A encoding, have high similarity (Heilmann et al., 1996). In addition, the *sig*B can also regulate the synthesis of PIA. When the *sig*B is activated, PIA synthesis decreases, resulting in less PIA-dependent biofilm formation (Valle et al., 2019). When TPCP was treated for 8 h and 16 h, the expression of *ica*A and *sig*B was increased at the same time (Figures 5C,E). This means that PIA synthesis by MRSA increases at this time, but the biofilm tends to be PIA-independent. This seems paradoxical. This contradiction is related to the fact that *sig*B is a global regulator of stress. When bacteria are exposed to environmental stresses such as antibiotics, reactive oxygen species, and acid–base imbalance, *sig*B expression is upregulated (Bonilla, 2020). This may be the reason why the *sig*B expression continued to increase after 8 and 16 h of dosing.

Furthermore, *sig*B is also involved in eDNA regulation (Valle et al., 2019). *sig*B inhibits eDNA degradation by decreasing the expression of thermonuclease regulatory genes. eDNA acts as the backbone of bacterial biofilms, promoting bacterial adhesion and biofilm formation (Li et al., 2021). It can also promote the transfer of drug resistance genes and increase bacterial resistance to antibiotics (Das et al., 2013). The TPCP significantly reduces the eDNA content of MSSA and MRSA after 24 h of treatment (Figures 4C,D). Research suggests that MSSA eDNA is primarily produced by programmed cell death (Campoccia et al., 2021). The *cid*A gene induces bacterial lysis and eDNA release by modulating murein hydrolase activity (Rice et al., 2007; Endres et al., 2022). The *cid*A expression in MRSA increased at 8 h after TPCP treatment and continuously increased at 16 h (Figure 5B). For this situation, we speculate that when MRSA is exposed to TPCP, its stress system response, *sig*B gene is abundantly expressed, a large amount of eDNA and PIA may be synthesized due to the overexpression of *cid*A and *ica*A, the result is to promote bacterial aggregation against the effects of TPCP. Finally, the continuous increase in *cid*A expression leads to the death of a large number of bacteria, which plays a role in inhibiting biofilm formation.

The accessory gene regulator (*agr*) and staphylococcal accessory regulator (*sar*) of *S. aureus*, a quorum-sensing system, play important regulatory roles in the expression of virulence factors and biofilm formation (Beenken et al., 2010; Jenul and Horswill, 2019). RNAIII transcripts are effectors of *agr* system that regulate the expression of *agr*-associated virulence and biofilm formation genes (Johnson et al., 2015) RNA-II encodes for four genes: *agr*B, *agr*D, *agr*C, and *agr*A. In Δ*agr*A mutant strains showed reduced ability to form biofilms (Aubourg et al., 2022). In our experiment, the expression of *agr*A increased 1.4-fold after 8 h of TPCP treatment and decreased 0.79-fold after 16 h of treatment (Figure 5A). SarA is the primary *sar* effector molecule (Dunman et al., 2001). *sar*A may also play an important role in the synthesis of nucleases and extracellular proteases. Nuclease and protease activities were significantly increased in sarA mutant strains (Beenken et al., 2010; Ramirez et al., 2020). The ability of Δ*sar*A strains to form films was significantly inferior to that of the wild type (Valle et al., 2003; Beenken et al., 2010; Ramirez et al., 2020). A decrease in PIA content was also observed in Δs*ar*A strains (Valle et al., 2003; Beenken et al., 2010). At 16 h, the expression of *sar*A was significantly higher than that of control-16 h, indicating that TPCP could not inhibit the expression of *sar*A. Although the expression of *sar*A decreased at MIC-16 h compared to MIC-8 h, the trend was not statistically significant. Considering the regulatory effect of *sar*A on proteases and nucleic acids and its ability to regulate the transcription of the collagen adhesion gene, we hypothesize that MRSA is stimulated by TPCP to favor biofilm formation pathways to resist the effects of TPCP. The inhibitory effect of TPCP on biofilms is not due to regulation of the *sar*A gene.

The results of this study demonstrated that TPCP has remarkable antibacterial activity and can disrupt biofilms of MSSA and MRSA. TPCP inhibited biofilm formation by reducing the secretion of EPS and eDNA. The regulation of biofilms by TPCP was closely related to the quorum sensing system. More conclusive evidence is needed for further experimental validation. In conclusion, TPCP has the potential to combat MRSA infection. However, in our experiments, we only evaluated the antibacterial effect of TPCP on standard strains and did not evaluate the effect on clinical isolates strains, which will be needed in the future. To verify the anti-infective potential of TPCP, we also need to investigate the effects of TPCP on virulence factors, energy metabolism, macromolecular substances and physiological activities, and pay attention to the effects on MSSA itself. For MRSA, the effects of TPCP on genes regulating drug resistance, resistance-related proteins and enzymes, and efflux pumps can be studied. And further in-depth molecular research is required to explain the precise antibacterial and antibiofilm processes. Validation of whether the antimicrobial activity of TPCP is the result of the synergistic effects of specific chemical markers or a combination of bioactive components will involve additional evaluation of the identified bioactive chemical markers in the plant extract. Besides, for drug discovery, more research concerning pharmaceutical properties should be conducted. For example, the pharmacokinetic studies would help to reveal the bioavailability and metabolic features of TPCP. The toxic studies, such as single-dose toxicity, repeated-dose toxicity, immunotoxicity, reproductive toxicity, and genotoxicity, would provide exact information of its safety.

## Data availability statement

The data that support the findings of this study are available from the corresponding author, JQ. Reasonable requests to access these datasets should be directed to junyuanqin@qq.com.

## Author contributions

JQ contributed to the concept of the study, did the experiments and wrote the manuscript. LY designed the work, drew and analyzed the data. XY, GL, CS, and CF did the experiments and analyzed data. FP and XF contributed to the concept of the study, review and edit the manuscript. CP contributed to the concept of the study and provide the funds. All authors contributed to the article and approved the submitted version.

## Funding

This study was funded by National Science Foundation of China (No. 82003879), the Key Project of Science and Technology Department of Sichuan Province (No. 20ZDYF3092); the regional Joint Fund of National Science Foundation of China: Study on the geo-herbalism of Medicinal Materials from Sichuan Tract (U19A2010); the Natural Science Foundation of China: Study on the property-efficacy Relationship of Traditional Chinese Medicine (No. 81891012); Multi-dimensional Evaluation of Traditional Chinese Medicine Resources with Characteristics in Southwest China (ZYYCXTD-D-202209); Multi-dimensional Evaluation and Product Development Innovation Team of Characteristic Chinese Medicine Resources in Southwest China (2022C001); Sichuan Provincial Youth Science and Technology Innovation Research Team (2020JDTD0022); and also supported by the National Scholarship Fund of China and Fund of the State Key Laboratory of Southwestern Chinese Medicine Resources.

## Conflict of interest

The authors declare that the research was conducted in the absence of any commercial or financial relationships that could be construed as a potential conflict of interest.

## Publisher’s note

All claims expressed in this article are solely those of the authors and do not necessarily represent those of their affiliated organizations, or those of the publisher, the editors and the reviewers. Any product that may be evaluated in this article, or claim that may be made by its manufacturer, is not guaranteed or endorsed by the publisher.
